# Chromodynamics of Cooperation in Finite Populations

**DOI:** 10.1371/journal.pone.0000270

**Published:** 2007-03-07

**Authors:** Arne Traulsen, Martin A. Nowak

**Affiliations:** Program for Evolutionary Dynamics, Department of Organismic and Evolutionary Biology, Department of Mathematics, Harvard University, Cambridge, Massachusetts, United States of America; University of Edinburgh, United Kingdom

## Abstract

**Background:**

The basic idea of tag-based models for cooperation is that individuals recognize each other via arbitrary signals, so-called tags. If there are tags of different colors, then cooperators can always establish new signals of recognition. The resulting “chromodynamics” is a mechanism for the evolution of cooperation. Cooperators use a secret tag until they are discovered by defectors who then destroy cooperation based on this tag. Subsequently, a fraction of the population manages to establish cooperation based on a new tag.

**Methodology/Principal Findings:**

We derive a mathematical description of stochastic evolutionary dynamics of tag-based cooperation in populations of finite size. Benefit and cost of cooperation are given by *b* and *c*. We find that cooperators are more abundant than defectors if *b/c* > 1+2*u/v*, where *u* is the mutation rate changing only the strategy and *v* is the mutation rate changing strategy and tag. We study specific assumptions for *u* and *v* in two genetic models and one cultural model.

**Conclusions/Significance:**

In a genetic model, tag-based cooperation only evolves if a gene encodes both strategy and tag. In a cultural model with equal mutation rates between all possible phenotypes (tags and behaviors), the crucial condition is *b/c* > (K+1)/(K−1), where *K* is the number of tags. A larger number of tags requires a smaller benefit-to-cost ratio. In the limit of many different tags, the condition for cooperators to have a higher average abundance than defectors becomes *b* > *c*.

## Introduction

The green beard effect was introduced by William D. Hamilton as a thought experiment in sociobiology: a gene that leads both to a visible tag (such as a green beard) and the tendency to help others with the same tag allows evolution of cooperation [1]–[11]. But if tags and behaviors evolve independently, then cheaters can undermine the system. Defectors might display the correct tag without providing any help. They will spread in the population, because they enjoy the support of cooperators without incurring the cost of cooperation. Thus, tag based cooperation seems to be a problematic idea.

Nevertheless, tags are abundant in social systems and provide good opportunities for distinguishing between in-group and out-group [12]. Tribal costumes or school uniforms are visible tags that indicate common grounds, possibly leading to cooperation. Fashionable clothing can be a secret sign among the few that are aware of the trend. Later, when the trend is picked up by many, the early adaptors switch to a new fashion. Wearing an uncomfortable tie can be a signal of conforming with social expectations. Cooperation can be based solely on these observable tags without the need of reputation or prior interactions (as is assumed in the framework of indirect reciprocity [13]).

There are also examples for tag based cooperation among animals. In the social amoeba Dictyostelium discoideum, single genes have been found that control both the tag and the corresponding helping behavior [14]. Because homophilic cell adhesion is responsible for both properties, cheating is not possible. The same mechanism seems to exclude cheating in green beard mechanisms found in conflicts of parental investment into offspring during pregnancy [15], [16]. Lizards cooperate based on the color of others, which serves as an indicator of male strategy [17]. There seem to be genetic constraints that do not allow disentangling throat color (and its recognition) from the behavioral strategy. Ant workers kill queens, who try to initiate reproduction, if they do not share a certain gene. This leads to tag based spiteful behavior [18]. Again, genetic constraints seem to exclude the possibility that ants create the odor cue that serves as a signal for the gene, but not the corresponding behavior. While the original green beard effect excludes defectors a priori, more general forms of tag based cooperation as the one described here consider situations in which individuals may have the tag, but not the corresponding behavior.

An example of tag-based cooperation on the internet are peer-to-peer networks [19]–[21]. In these networks, computer programs and files are shared among participants. A cooperator is someone who contributes his own high quality files, whereas defectors just download from the community. Often it is not easy to assess the quality of these networks from the outside and different mechanisms are applied to prevent defectors from joining networks, such as restricting new membership to acquaintances of old members. However, in the long run such mechanisms can fail and cooperation might break down. Then new networks have to be initiated by the cooperators.

There have been several theoretical approaches to tag based cooperation. Riolo et al. [5] have introduced a model with a continuum of tags, but which does not include the possibility of cheating against somebody who uses the same tag [6]. The basic aspects of this model can be understood by considering a system with only two tags [7], [8]. Axelrod et al. [9] have shown that tags can lead to cooperation in the presence of cheaters in structured populations. Jansen and van Baalen [10] have considered tag based cooperation in a system with one gene for the tag and a second gene for the strategy. In their spatial model, tags lead to high levels of cooperation even if no cooperation is expected based on the population structure alone.

Cooperators might recognize each other by a secret handshake [22]. Once defectors find out about this handshake, it loses its value. Cooperators must establish a new handshake. There is a permanent race between cooperators and defectors: cooperators are trying to encode new handshakes, while defectors attempt to break their code. The crucial question is under which conditions can cooperators run faster than defectors? Here, we will answer this question based on an analysis of a model similar to that of Jansen and van Baalen [10], but formulated for finite, well-mixed populations. In contrast to the model of Jansen and van Baalen where the coexistence of many tags is possible, the analytical description of our model considers only two tags at a time and tag diversity is only reflected by the mutation rates.

**Figure 1:**
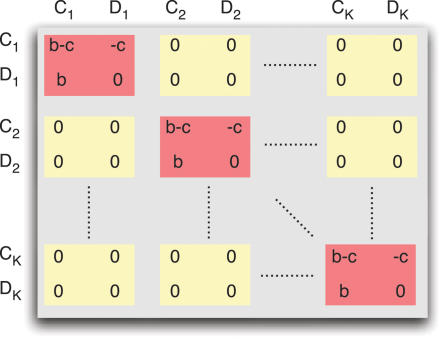
Payoff matrix for chromodynamics of cooperation. Interactions that lead to nonzero payoffs only occur between individuals using the same tag. For a given tag, defectors always dominate cooperators. By continuously changing the ’secret handshake’ ( = tag), cooperators can run away from defectors. For a cultural model, it turns out if *b/c*>(*K*+1)/(*K*−1), then cooperators can run faster than defectors.

In our model, the number of possible tags is given by *K*. Individuals interact with others who have the same tag. For each tag, *i* = 1,…,*K* there are cooperators, *C_i_*, and defectors, *D_i_*. In total there are *2K* different strategies. Cooperators help all others with the same tag at a cost, *c*, for the donor and a benefit, *b*, for the recipient. The payoff matrix is shown in Figure 1. This payoff matrix has the property that the sum of the diagonal elements is the sum of the offdiagonal elements. However, not all subgames have this property: For example, the *2×2* game between defectors and cooperators with different tags does not have this property. While our analysis does not rely on the particular choice of this payoff matrix, it allows us to write our results in a form that is easy to interpret.

We consider evolutionary game dynamics in finite populations of size *N* including the effects of selection, mutation and random drift [23]. The population is well mixed. As update rule we use pairwise comparison [24]–[27]. In each time step, two individuals are chosen at random. The first individual adopts the strategy of the second individual with probability (1+*e* ^+*β*(*π*_1_−*π*_2_)^)^−1^. Here *π*_1_ and *π*_2_ denote, respectively, the payoffs for the first and second individual. The parameter β measures the intensity of selection. It behaves like an inverse temperature in statistical physics [27]. For *β*→∞, the process always follows the gradient of selection. The case of weak selection is given by *β* << 1/N [28]. This stochastic process is very similar to the frequency dependent Moran process [29], [30]. For weak selection, the two processes have the same fixation probabilities.

**Figure 2:**
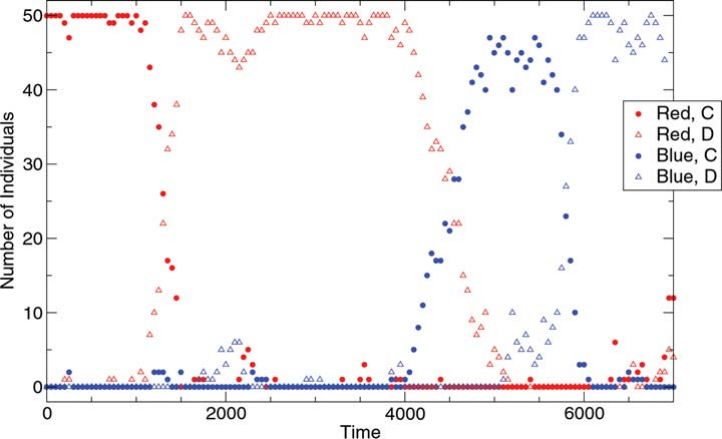
Evolutionary chromodynamics in finite populations. The red cooperator population is invaded by red defectors at *t*≈1200. At *t*≈4500, cooperation is established based on blue tags. Blue defectors invade at *t*≈6000. The time unit is given by one individual learning event (pairwise comparison). For example, after *t* = 5000 each individual had *100* learning events on average. The following parameters are used: population size *N = 50*, intensity of selection *β = 1.0*, cost of cooperation *c = 0.5*, benefit from cooperation *b = 1.0*, mutation rate *u = 0.01*.

With a small probability an individual ‘mutates’ to adopt a randomly chosen strategy. Computer simulations of the resulting mutation selection process are shown in Figure 2.We start with a population of cooperators using tag *i* = 1. After some time, defectors emerge who uses the same tag and can therefore exploit the cooperators. The whole population turns to defection. Eventually, a cooperator arises with a different tag. As long as only a single cooperator with a different tag is present, it is neutral.

But as soon as neutral drift leads to more cooperators with this tag, they become advantageous. These cooperators dominate the population until they are again ‘discovered’ by defectors, who then destroy cooperation based on this tag. Cooperators with a new tag arise and so on. Reminiscent of a ‘red queen’ mechanism [31] cooperators have to change their tag continuously to free themselves from defectors. This concept is called ‘chromodynamics’ by Jansen and van Baalen [10], [32].

## Results and Discussion

The analysis of our model is very different in finite populations differs considerably from infinite populations. When a small fraction of defectors of every possible tag is present, this hampers the evolution of cooperation in our model, as there are no niches where invading cooperators can thrive. This happens when the population is very large and extinction takes a long time. In finite populations, this situation occurs if the mutation rates between the different strategies are large and all types are continuously produced. Nonetheless, our numerical simulations show that cooperation can evolve in finite populations even for high mutation rates if the benefit to cost ratio is sufficiently high. An analytic calculation of evolutionary chromodynamics in finite populations is possible in the limit of small mutation rates [33], [34]. In this case, we can describe the evolutionary dynamics by transitions between homogeneous states. There are four types of relevant transitions: (i) from *C_i_* to *D_i_*, (ii) from *C_i_* to *D_j_*, (iii) from *D_i_* to *C_i_*, and (iv) from *D_i_*to *C_j_*. In the Appendix, we show how to calculate these transition rates. For weak selection (small *β*), the transition rates are given by 
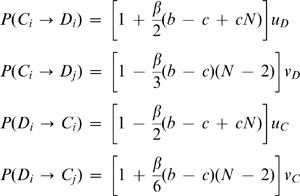
1The parameters *u_C_* and *u_D_* denote the mutation rates changing the strategy, but not the tag. The parameters *v_C_* and *v_D_* denote the mutation rates of changing the strategy and the tag.

The system will spend more time in cooperator states, if the sum of the transition rates into cooperator states exceeds the sum of the transition rates out of cooperator states, 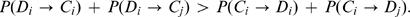
2In this case cooperators are risk-dominant over defectors [29], [35]. This inequality leads to
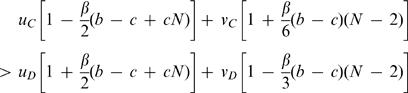
3In the limit of vanishing selection, *β*→0, we obtain 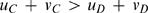
4In this limit, any potential asymmetry in the mutation rates decides which strategy is risk dominant and selection terms have no influence. To perform a meaningful weak selection analysis, we must therefore assume that the mutation rates are symmetric in the sense that
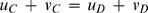
5Using eq. (5), inequality (3) leads to
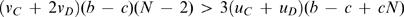
6For large populations, we obtain 
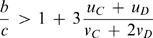
7Note that this condition is based on the constraint given by eq. (5). For *u = u_C_ = u_D_* and *v = v_C_ = v_D_*, inequality (7) leads to
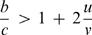
8Now let us make some specific assumptions about the relative magnitude of the mutation rates *u* and *v*.

At first, we consider a genetic toy model where the genotype is given by a bit string of length *L+1*. One bit encodes the strategy; *0* denotes cooperation, and *1* denotes defection. *L* bits encode the tag. Hence, there are 2*^L^* possible tags. With a mutation rate of *µ* per bit, we have *u = μ(1−μ)^L^*and *v = μ^2^L(1−μ)^L−1^* (neglecting double mutations in the tags). For these mutation rates we obtain
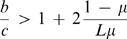
9This inequality suggests that a very large benefit to cost ratio is needed for the case of a small mutation rate, *µ*, which is required for the validity of our analytical approximation.

**Figure 3:**
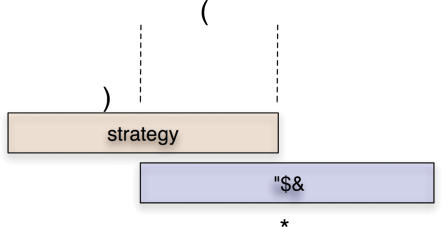
Consider a pleiotropic gene that encodes both strategy and tag. The first *n* bits encode the strategy according to a parity rule: if the sum of the first *n* bits is even, the strategy is cooperation, otherwise it is defection. The last *m* bits encode the tag. Each sequence encodes a different tag. Hence there are 2^m^ possible tags. There is an overlapping region of *L* bits which affect both the strategy and the tag. This setup allows evolution of tag based cooperation if *b/c>(2n−L)/L* is fulfilled.

As a second model, we consider a pleiotropic gene given by a bit string of certain length. This gene encodes both the behavioral strategy and the tag. The parity of the first *n* bits determines the strategy: the genotype encodes cooperation if there is an even number of *1s*; the genotype encodes defection if there is an odd number of *1s*. We use the parity because (i) any mutation in the *n* bits changes the strategy and (ii) the mutation rates in both directions are equally fast. The last *m* bits determine the tag. We have 2*^m^* possible tags. We assume that the two regions have an overlap of *L* bits, see Fig. 3. A mutation in the strategy that does not change the tag occurs with rate *u = (n−L)μ*. The mutation rate that simultaneously changes tag and strategy is *v = Lμ*, neglecting terms of the order of *μ* ^2^. This leads to the condition 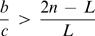
10The critical benefit to cost ratio is small as long as *L* is a sizable fraction of *n*.

**Figure 4:**
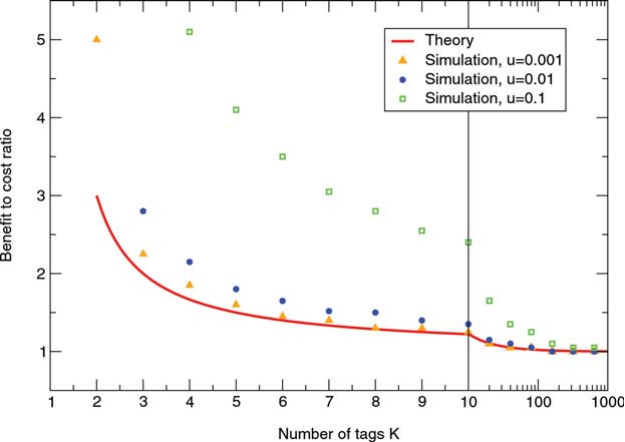
In a model with *2K* phenotypes consisting of a pair of strategy and tag, cooperation evolves depending on the benefit to cost ratio. For small mutation rates, the critical benefit to cost ratio for evolution of cooperation is given by *b/c>(K+1)/(K−1)* (red line). If the benefit to cost ratio exceeds this critical value, then cooperators are more abundant than defectors averaged over time. With increasing mutation rates, the populations become more mixed which favors defectors. Hence, the critical benefit to cost ratio increases with a higher mutation rate, as shown for *u = 0.01* and *u = 0.001*. In all cases, the critical benefit to cost ratio decreases with the number of tags *K* and converges to *1* for *β*→∞. The following parameters are used: population size *N = 100*, intensity of selection *β = 0.1*, cost of cooperation *c = 0.2*, averages over 10^8^ time steps.

Finally, let us consider a system with *2K* phenotypes consisting of a pair of strategy and tag. The mutation rate between all phenotypes is constant and given by µ. Therefore, we have *u = μ* and *v = (K−1)μ*. This yields
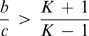
11At the very least, two different tags are needed. For *K = 2*, the crucial condition for risk dominance of cooperation is *b/c*>3. If there are many tags, *K*>>1, we only require *b/c*>1. This is the minimum condition for any evolution of cooperation, see Fig. 4. If *b* does not exceed *c* then cooperation does not generate an overall benefit. Thus, *b/c*>1 implies that cooperation evolves for free. For large *K*, cooperators have always higher average abundance than defectors. We call this limit ‘altruistic freedom’.

Jansen and van Baalen [10] essentially assume that the tags and strategies are encoded by different genes. In the context of our model, this leads back to a condition similar to [9]. For this choice of mutation rates, the critical benefit to cost ratio is independent of the number of tags *K* and becomes very large for small mutation rates. Therefore, in the model of Jansen and van Baalen [10] cooperators are not expected to dominate in a well-mixed population. Their model relies on spatial structure. In a genetic model, it seems natural to assume that tag and behavior are encoded by different genes. In this case, the mutation rates of Jansen and van Baalen apply and evolution of cooperation based on tags requires the help of spatial structure.

For a cultural model, which is based on learning and imitation of strategies, it not unreasonable to assume that each phenotype is given by a combination of tag and behavior and that mutations among phenotypes occur at equal rates. For example, someone who has realized that cooperation based on a ‘red tag’ is no longer possible and therefore behaves as a ‘red defector’, might have the idea to establish cooperation based on a ‘blue tag’ and hence ‘mutate’ from red defection to blue cooperation. Later, another red defector might mutate to become a blue defector. It is conceivable that both mutation events occur with similar rates. If there is a roughly constant mutation rate among phenotypes, then tag based models can facilitate the evolution of cooperation even in well-mixed (non-spatial) populations.

There is a simple intuitive way to justify our main result, eq. (11). The evolutionary dynamics of our model are determined by two different types of transitions. The first type describes a competition between cooperators and defectors who use the same tag; the resulting game is described by the standard Prisoner's Dilemma payoff matrix
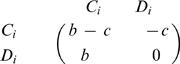
12The second type of transition describes a competition between cooperators and defectors who use different tags; in this case the payoff matrix is given by
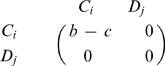
13If there are K many tags, then the second type of transition has the chance to occur *K-1* times as often as the first type in our cultural model. We can add up the two payoff matrices after multiplying the second matrix by *K-1*. This yields 
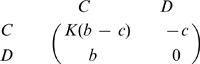
14Cooperators are risk dominant over defectors if the sum of the entries in the first row exceeds the sum of the entries in the second row [35]. We obtain *K(b−c)−c>b* which leads to condition (11).

It should be noted that tag-based cooperation in well mixed populations is different from tag-based cooperation in structured populations. In well mixed populations, cooperation based on tags can only dominate for a limited time [5], [7], [10], leading to “Tides of tolerance” [36]. In our case, we discuss the condition under which the average abundance of cooperators is higher than the average abundance of defectors. In spatial systems, persistent cooperation based on tags is possible [8]–[10]. But in spatial models (or on graphs) tags are not necessary for the evolution of cooperation [37], [38].

The various mechanisms for the evolution of cooperation include kin selection [1], [39]–[44], group selection [45]–[48], direct reciprocity [49], [50], indirect reciprocity [51]–[54], and network reciprocity [37], [38], [55]–[59]; for a review of these mechanisms see [35]. Tag based models could provide another mechanism for evolution of cooperation. In this paper, we have derived a simple condition for the evolution of cooperation by tags. The benefit-to-cost ratio of the altruistic act, *b/c*, has to exceed the ratio 1+2*u/v* where the mutation rate *u* changes only the strategy and the mutation rate *v* changes strategy and tag simultaneously. In a genetic model, cooperation evolves only if a gene encodes both strategy and tag. In a cultural model where the different types are characterized by tag and strategy, the ratio becomes (*K*+1)/(*K*−1) where *K* is the number of different tags. For *K* = 2 tags we need *b/c*>3. For many different tags, *K*>>1, we only need *b/c*>1. If there is a large number of tags, cooperation evolves for free in a cultural model. Chromodynamics with a multitude of different colors ( = tags) can lead to altruistic freedom.

## Materials and Methods

Consider a game between two strategies, A and B, given by the general payoff matrix 
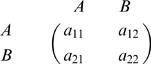
15If there are *i* many A players and *N-i* many B players, then the payoffs for A and B are given by 
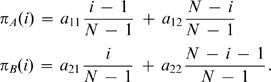
16Using pairwise comparison updating [27], the probability that the number of A individuals changes from *i* to *i*±1 is 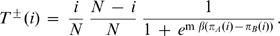
17The probability that a single individual of type A takes over a population of type B is
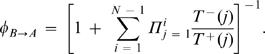
18For strong selection (large *β*), the fixation probabilities can be computed from this formula or from a closed expression that is a very good approximation for this formula [27]. For weak selection, *β*<<1, the fixation probabilities reduce to
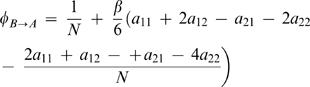
19This is identical to the corresponding result of the frequency dependent Moran process [29]. From eq. 19, we can calculate the transition rates given in the text.

As an example, consider the transition from defectors to cooperators of a different tag. In this case, we have *a*_11_ = *b−c* and *a*_12_ = *a*_21_ = *a*_22_ = 0, which yields 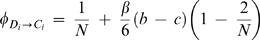
20Eq. (1) can be obtained by choosing the appropriate entries of the payoff matrix for *a_ij_*. The transition rates of eq. (1) represent the transition probabilities multiplied with population size and mutation rate.
